# Ursodeoxycholic acid in the prevention of gallstones in patients subjected to Roux-en-Y gastric bypass^1^

**DOI:** 10.1590/s0102-865020190010000009

**Published:** 2019-02-14

**Authors:** Francisco Heine Ferreira Machado, Heladio Feitosa de Castro, Rodrigo Feitosa de Albuquerque Lima Babadopulos, Hermano Alexandre Lima Rocha, José Lima de Carvalho Rocha, Manoel Odorico de Moraes

**Affiliations:** I PhD, Department of Surgery, Universidade Federal do Ceará (UFC), Fortaleza-CE, Brazil. Intellectual, conception and design of the study, critical revision, final approval.; II MD, Department of Surgery, UFC, Fortaleza-CE, Brazil. Analysis and interpretation of data, final approval.; III MD, Hospital Geral Dr César Cals de Oliveira, Fortaleza-CE, Brazil. Analysis and interpretation of data, final approval.; IV PhD, Community Health Department, UFC, Fortaleza-CE, Brazil. Analysis and interpretation of data, final approval.; V PhD, UNICHRISTUS, Fortaleza-CE, Brazil. Analysis and interpretation of data.; VI PhD, Full Professor, Department of Farmacology, UFC, Fortaleza-CE, Brazil. Manuscript writing, critical revision, final approval.

**Keywords:** Obesity, Bariatric Surgery, Gallstones, Ursodeoxycholic Acid

## Abstract

**Purpose:**

To evaluate the contribution of ursodeoxycholic acid (UDCA) in the first 12 months after Roux-en-Y gastric bypass in the prevention of gallstone formation.

**Methods:**

A community-based clinical trial was conducted. A total of 137 patients were included in the study; 69 were treated with UDCA, starting 30 days after the surgery, at a dose of 150 mg twice daily (300 mg/day) over a period of 5 consecutive months (GROUP A), and 68 were control patients (GROUP B). The patients were followed-up, and ultrasonography was performed to determine the presence of gallstones at various times during follow-up. Demographic, anthropometric and comorbid indicators were obtained. The data were subjected to normality tests and evaluated using appropriate tests.

**Results:**

Patients did not differ in their baseline characteristics. Of the 69 patients who used UDCA, only one patient developed cholelithiasis (1%), whereas 18 controls (26%) formed gallstones (OR = 24.4, p <0.001). Also, other factors were found not to influence the formation of calculi, such as pre-operative or postoperative hepatic steatosis or diabetes (p = 0.759, 0.468, 0.956).

**Conclusion:**

The results demonstrated that patients who did not use UDCA showed a 24.4-fold greater probability of developing cholelithiasis.

## Introduction

 Morbid obesity has reached epidemic proportions in the Western world and has high human and financial costs in the United States. It is one of the most deadly diseases in the world(1). This has resulted in a dramatic increase in surgeries, and currently more than 140,000 bariatric surgeries are performed annually in the United States alone^1^.^ ^ In Brazil, data collected by the Family Budget Survey 2002-2003 of the Brazilian Institute of Geography showed that 38.8 million Brazilians over 20 years old were overweight, and 11% of them were classified as obese.

 During weight loss after bariatric surgery, the risk of developing gallstones increases and accordingly the development of complications of cholelithiasis as well. Due to the increase in gallstone disease, some centers routinely recommend prophylactic cholecystectomy. However, this practice remains controversial because not all patients develop cholelithiasis after bariatric surgery and cholecystectomy during bariatric surgery can be a difficult procedure, increasing the risks of iatrogenic bile duct injury. The alternative of prophylactic cholecystectomy without cholelithiasis is inadvisable. There are three serious complications: acute biliary pancreatitis, with morbidity and significant mortality, with biliary pancreatitis being more severe than alcoholic pancreatitis, non-suppurative cholangitis and suppurative cholangitis, with 100% mortality when only clinical treatment is performed^2^ ^,^ ^3^.

 Ursodiol (ursodeoxycholic acid, UDCA) is found naturally in bile acids at less than 5% in humans and at a very high percentage in bears. Following oral administration, it is absorbed, conjugated in the liver with glycine or taurine, and excreted in the bile, and conjugated UDCA enters the enterohepatic recirculation. The serum half-life is approximately 100 h. With daily administration, over a long time, UDCA constitutes 30-50% of the bile acids. UDCA lowers cholesterol concentration by reducing liver cholesterol secretion. UDCA is used for dissolution of small calculi in the gallbladder in patients with asymptomatic cholelithiasis, in those symptomatic who refuse to undergo cholecystectomy or in those with a high surgical risk. UDCA is more effective than chenodeoxycholic acid and results in a decrease in stone formation of 32% compared to 2% for placebo) by increasing the solubility of cholesterol and decreasing cholesterol saturation in the bile^3^ ^,^ ^4^.

 At a dose of 10 mg/kg/day for 12-14 months, dissolution was over 50% in patients with small non-calcified calculi (<5-10 mm). It is also effective in the prevention of gallstone disease in obese patients who undergo therapy with rapid weight loss. Studies show that UDCA at a dose of 13-15 mg/kg/day helps patients with early biliary cirrhosis, reducing liver function abnormalities and improving liver histology. UDCA is virtually free of adverse side effects. It is uncommon for bile salts to cause diarrhea^4^. 

 Considering that UDCA is effective in the dissolution of calculi and has already been recommended to reduce the incidence of stones, the aim of this study was to determine the effectiveness of UDCA in preventing the formation of calculi in the postoperative period of patients subjected to bariatric surgery, during the rapid weight loss phase.

## Methods

 The project was submitted to the Research Ethics Committee of the Universidade Federal do Ceará, in compliance with the Nacional Council o Health, Resolution 466/12. Data collection only took place after approval at Plataforma Brasil, where the project was accepted under Protocol No. 1.024.054, de 26/03/2015. The study was registered in REBEC (Rede Brasileira de Ensaios Clínicos) under number U1111-1205-5877, retrospectively. 

###  Study type and population 

 We conducted a comparative, prospective, community-based study consisting of a comparison of two groups: GROUP B that did not take UDCA and GROUP A that did take UDCA, which was started 30 days after the surgery (150 mg/day given twice daily) over a period of 5 consecutive months. The patients had a body mass index (BMI) greater than or equal to 40 kg/m², or they had a BMI greater than or equal to 35 kg/m² with associated severe comorbidities who failed on conservative treatment. All patients underwent a complete evaluation performed by a multidisciplinary team, and were included paired by order of inclusion. The surgical technique was strictly the same for two groups. CONSORT guidelines were followed.

###  Study site 

 This study was performed in Fortaleza-CE, from April 2015 to August 2016, in two clinics providing service in non-governmental bariatric surgery: Obesity Center and Monsenhor Bruno Clinic, which are duly authorized by the Health Department of the State of Ceará, within the established criteria By the Unified Health System to provide a High Complexity Assistance Service, in accordance with Ministerial Order No. 425/GM/MS (March 19, 2013) of the Brazilian Ministry of Health^5^. 

###  Study sample 

 The population was composed of 137 patients who underwent bariatric surgery by videolaparoscopy from January 2014 to December 2015. They were allocated into two groups: 68 patients in GROUP B and 69 patients in GROUP A, according to the clinic of reference, and data were collected in medical records.

#### Inclusion criteria

**Figure 1:**
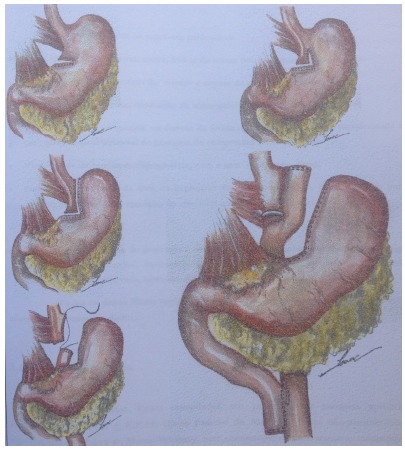
Capella Operation - Gastric Bypass (without ring) - Surgical Times: Gastric Bag: 30-40cm³, Food Strap: 120-140cm (140cm: in the super-obese), Biliary Handle: 80-100cm (100cm: diabetic patients).

 The following inclusion criteria were used: patients of both sexes who underwent bariatric surgery using the Roux-en-Y gastric bypass technique with videolaparoscopy from January 2014 to December 2015; over 18 years old; BMI greater than or equal to 40 kg/m^2^ or BMI greater than or equal to 35kg/m^2^ but with associated comorbidities according to criteria established by the National Institutes of Health Consensus Development Conference Statement of 1991^6^; and no previous gallstones as evidenced by preoperative abdominal ultrasonography. The surgical technique consists of making a gastric pouch of 30-40ml, intestinal loop of 120 cm and biliary loop of 80-100cm (Fig. 1). All patients underwent Roux-en-Y gastric bypass by videolaparoscopy by the same surgical team, to reduce possible distortions or biases resulting from the treatment adopted.

#### Exclusion criteria

 Excluded from the study were patients who underwent cholecystectomy prior to bariatric surgery, patients with gallstones, participants in other investigations with investigative drugs, pregnant women, those unable to sign and informed consent form, patients without clinical conditions to participate in the study, and those who refused to participate. Regarding the therapy, patients who did not use UDCA according to the protocol or with a follow-up period of less than six months postoperatively, or who had previously used UDCA, in any way, as recorded in the medical records.

###  Statistical analysis 

 The following variables were collected: distribution of the sample for analysis according to the data of the research site, nationality, gender and marital status; distribution of the sample for analysis according to age, initial weight and weight 6 months after surgery, height, initial BMI, current BMI, difference in BMI, and weight difference, distribution of the sample for analysis according to the main comorbidities evaluated before surgery and 6 months after surgery, namely hepatic steatosis (and whether mild, moderate or severe), gastroesophageal reflux (GERD), gastritis, systemic arterial hypertension, cardiovascular disease, dyslipidemia, diabetes mellitus 2, sleep apnea, osteoarthritis, infertility and gallstones. Abdominal ultrasonography was performed to determine the presence of gallstones at during follow-up in all patients, six months after the surgery. 

 Categorical data were presented as percentages and counts and the numerical data as means and standard error of means. Normality tests were performed for numerical variables. Depending on the normality of the variables, ANOVA or the Mann-Whitney test was performed, as appropriate. The chi-square test was used for categorical data. The McNemar test was used for paired before and after variables. Odds ratios were calculated to measure the association of the determinants with the main outcome. p < 0.05 was considered significant. SPSS software (Statistical Package for the Social Sciences), v23, SPSS, Inc. was used for the analysis and evaluation of the data obtained.

## Results

 In Group A, 18 (26.1%) patients were male, and in Group B, 27 (39.7%) were male (p = 0.090). The mean age of the cases was 34.18 (SD = 13.36) in group A, and the mean age was 35.91 (SD = 10.28) in group B. There was also no difference between the groups for anthropometric measures, with mean weights of 112 and 116 in the test and control groups, respectively (p = 0.377), with the same pattern for the other variables, such as hepatic steatosis (p = 0.361). Among the comorbidities, there was a higher prevalence of sleep apnea and GERD in the test group (p = 0.004 and 0.020, respectively) (Table 1). 100% of patients complied with the use of medication. 

**Table 1:** Biological profile and previous pathologies of patients who used and did not use UDCA (Obesity Center).

		Site				
		MB Clínic		Obesity Center		
		MEAN	SEM	MEAN	SEM	p
Age		34.18	13.36	35.91	10.28	
Initial weight		112.00	21.00	116.00	23.00	0.274
Initial height		1.63	0.09	1.66	0.10	0.147
Initial BMI		41.80	5.00	42.20	5.80	0.718
Current BMI		27.60	3.80	27.60	3.60	0.980
		**N**	**%**	**N**	**%**	**p**
Gender	Male	18	26.1%	27	39.7%	0.090
Hepatic steatosis before		58	84.1%	53	77.9%	
Degree of steatosis	No	11	15.9%	15	22.1%	0.739
	Mild	24	34.8%	19	27.9%	
	Moderate	24	34.8%	23	33.8%	
	Severe	10	14.5%	11	16.2%	
GERD before		46	66.7%	32	47.1%	0.020
Gastritis before		22	31.9%	15	22.1%	0.195
Hypertension before		40	58.0%	33	48.5%	0.268
Cardiovascular disease before		3	4.3%	1	1.5%	0.317
Dyslipidemia before		43	62.3%	36	52.9%	0.267
Diabetes II before		21	30.4%	25	36.8%	0.433
Sleep apnea before		66	95.7%	54	79.4%	0.004
Osteoarthritis before		64	92.8%	62	91.2%	0.734
Infertility before		7	10.4%	1	1.5%	0.027

 The medication showed a strong association with the development of cholelithiasis, as can be seen in Table 2. Of the patients in GROUP A, 68 (98.5%) did not develop gallstones, where only 1 (1.4%) patient formed gallstones. On the other hand, 50 (73.5%) patients in GROUP B did not develop gallstones, while 18 (26.4%) did, showing a statistical significance in the determination of cholelithiasis (OR = 24.4, 95% CI = 3.1-189.4, p < 0.001).

**Table 2:** Distribution of results in the postoperative period with comparison of the sample between patients in GROUP A and those in GROUP B.

		Postoperative cholelithiasis						
		No		Yes				
		N	%	N	%	OR	95%CI	P
Use of medication	Yes	68	98.5%	1	1.4%	24.4	3.1-189.4	<0.001
	No	50	73.5%	18	26.4%			

 Other factors were tested for their influence on the formation of calculi, including anthropometric measurements before and after surgery, comorbidities such as hepatic steatosis before and after surgery, as well as occurrence of diabetes and hypertension, among others, but no other factor was associated with the formation of gallstones other than UDCA use (Table 3).

**Table 3:** Comparisons between patients who formed gallstones in the postoperative period according to anthropometric and comorbidities.

		Postoperative cholelithiasis						
		No		Yes				
		Mean	SEM	Mean	SEM			p
Age		35.01	12.28	35.25	9.69			0.943
Initial weight		114.00	21.00	116.00	30.00			0.664
Initial height		1.64	0.09	1.65	0.10			0.657
Initial BMI		41.00	4.80	42.30	8.50			0.843
Weight after surgery		75.00	13.00	76.00	19.00			0.871
Current BMI		27.70	3.50	27.40	5.20			0.767
Weight difference		38.90	11.10	40.70	13.70			0.528
BMI difference		14.30	3.30	14.90	4.50			0.536
		**N**	**%**	**N**	**%**	**OR**	**IC95%**	**p**
Hepatic steatosis before	Yes	96	81.4%	15	78.9%	1.16	0.26-2.84	0.759
	No	22	18.6%	4	21.1%			
Degree of steatosis	No	22	18.6%	4	21.1%	-	-	0.573
	Mild	35	29.9%	8	42.1%			
	Moderate	43	36.8%	4	21.1%			
	Severe	18	15.3%	3	15.8%			
Hepatic steatosis after	No	113	95.80%	19	100%	1.17	1.09-1.25	0.359
	Yes	5	4.20%	0	0%			
GERD before	No	54	46.2%	5	26.3%	2.36	0.81-7.09	0.106
	Yes	63	53.8%	14	73.7%			
GERD after	No	66	98.5%	1	100.0%	1.02	0.98-1.05	0.902
	Yes	1	1.5%	0	0.0%			
Gastritis before	No	88	75.2%	12	63.2%	1.77	0.64-4.92	0.269
	Yes	29	24.8%	7	36.8%			
Gastritis after	No	65	95.6%	1	100.0%	1.02	0.98-1.05	0.829
	Yes	3	4.5%	0	0.0%			
Hypertension before	No	54	46.2%	10	52.6%	1.29	0.49-3.42	0.600
	Yes	64	53.8%	9	47.4%			
Hypertension after	No	66	97.0%	1	100.0%	1.02	0.98-1.05	0.861
	Yes	2	3.0%	0	0.0%			
Cardiovascular disease before	No	114	96.6%	19	100.0%	1.17	1.09-1.25	0.413
	Yes	4	3.4%	0	0.0%			
Cardiovascular disease after	No	114	96.6%	19	100.0%	1.17	1.09-1.25	0.413
	No	67	98.5%	1	100.0%	1.02	0.98-1.05	0.902
	Yes	1	1.5%	0	0.0%			
Dyslipidemia before	No	51	43.6%	7	36.8%	1.33	0.48-3.61	0.581
	Yes	66	56.4%	12	63.2%			
Dyslipidemia after	No	49	73.1%	1	100.0%	1.02	0.98-1.06	0.546
	Yes	19	26.9%	0	0.0%			
Diabetes II before	No	79	67.5%	12	63.2%	1.21	0.44-3.33	0.708
	Yes	39	32.5%	7	36.8%			
Diabetes II after	No	64	97.0%	1	100.0%	1.02	0.98-1.05	0.860
	Yes	3	3.0%	0	0.0%			
Sleep apnea before	No	12	10.2%	5	26.3%	3.12	0.96-10.20	0.062
	Yes	106	89.8%	14	73.7%			
Sleep apnea after	No	36	53.7%	1	100.0%	1.03	0.97-1.08	0.356
	Yes	32	46.3%	0	0.0%			
Osteoarthritis before	No	9	7.7%	2	10.5%	1.41	0.28-7.09	0.674
	Yes	109	92.3%	17	89.5%			
Osteoarthritis after	No	38	55.9%	1	100.0%	1.03	0.97-1.08	0.565
	Yes	30	44.1%	0	0.0%			
Infertility before	No	109	94.0%	18	94.7%	1.15	0.13-10.0	0.895
	Yes	7	6.0%	1	5.3%			
Infertility after	No	62	95.4%	1	100.0%	1.02	0.98-1.05	0.826
	Yes	3	4.6%	0	0.0%			
Hernia postoperative	No	114	96.6%	19	100.0%	1.17	1.09-1.25	0.413
	Yes	4	3.4%	0	0.0%			

## Discussion

 In 1980, it was routine to recommend cholecystectomy for patients undergoing bariatric surgery because of the reported high incidence (30%) of symptomatic cholelithiasis. Currently, there is no consensus on the treatment of asymptomatic cholelithiasis in patients undergoing surgery for rapid weight loss, and thus, further investigations in the form of randomized, prospective studies are needed to more clearly define the indications for cholecystectomy at the time of weight loss surgery^7^ ^,^ ^8^.

 A pilot study confirmed the high incidence of gallstones (71% of patients evaluated) associated with rapid weight loss in patients who underwent gastric bypass surgery^9^ ^,^ ^10^. The migration of gallstones into the common bile duct, in obese patients who have undergone bariatric surgery can lead to a complex and severe complication. That is why in these patients who are at higher perioperative risk, minimal treatment of the calculi in the common bile duct is very important. After gastric bypass, endoscopic access to the biliary tree is very difficult, but it is possible through a laparoscopy-assisted transgastric anterograde approach^11^. Also an option to approach Vater’s papilla endoscopically in patients with a long afferent jejunal loop is to perform a surgical access through the stomach by gastrostomy, or jejunum, through a jejunostomy at the angle of Treitz (Fig. 1)^12^. Double balloon ERCP can also be another way to access gallstones into the common bile duct^13^.

 Prophylactic medication with UDCA in the prevention of gallstone formation after gastric bypass surgery may be proposed because of its effectiveness, as was shown in a systematic review and in this study^14^. Worobetz^15^ published a double-blind study in which one group received a placebo and the other group UDCA to study the prevention of gallstone development in 29 morbidly obese patients who underwent bariatric surgery. Six of the 14 placebo-treated patients (43%) developed gallstones. None of the 10 patients treated with UDCA formed gallstones.

 In clinical practice, it has been observed that during rapid weight loss¸ the formation of gallstones begins to occur after only 4 weeks. Prophylactic treatment with UDCA at 600 mg/day in the six-month period after bariatric surgery has been advocated by researchers in preventing gallstones or biliary sludge, factors responsible for developing severe complications such as biliary pancreatitis^9^ ^,^ ^16^ ^,^ ^17^.

 In this study, the group of patients who did not use UDCA were 24.1 times more likely to form cholelithiasis, with significant statistical significance (OR = 24.4, 95% CI = 3.1-189.4, p<0.001). It was also checked if there were other factors that could be confounding the result, but it was identified that no other factors were associated with the outcome.

 In addition to the already reported benefits, it has been highlighted in the literature that UDCA, a hydrophilic bile acid, may block the progression of non-hepatic fatty liver disease to non-alcoholic steatohepatitis by protecting hepatocytes through handling bile salts in mitochondrial trauma, anti-apoptotic signaling pathway, anti-inflammatory, antioxidant, immunomodulatory function, anti-fibrotic properties, and is being widely used in liver diseases^18^ ^-^ ^22^.

 It is worth noting that the administered doses varied among studies, and even so there was a significant effect found. Also, no studies with longer duration were found, neither with drop out of the medication, to estimate time of treatment with benefit.

###  Limitations 

 The study was conducted through a community clinical trial, which could have introduced selection bias and confusion among participants. However, these were processed during statistical analysis, which identified that the patients were fully comparable and that there was no confounding in the association of the main outcome, as well as ensuring that the surgical intervention was strictly the same in the two groups. Also, we didn´t used a placebo in the control group.

## Conclusions

 The effectiveness of UDCA in preventing the formation of gallstones in patients in the postoperative period of bariatric surgery when used during the rapid weight loss phase. Considering other findings in the literature and the risks of cholelithiasis complications, it is concluded that UDCA therapy may be beneficial if applied on a large scale. It is hoped that with advances with specific drugs, through research in clinical pharmacology and genetic studies, the treatment of morbidly obese patients will become more efficient.

## References

[1] Di Palma JA (2012). Current diagnosis & treatment gastroenterology, hepatology,
& endoscopy. Gastroenterology.

[2] Mason EE, Renquist KE (2002). Gallbladder management in obesity surgery. Obes Surg.

[3] De Oliveira CIB, Chaim EA, Da Silva BB (2003). Impact of rapid weight reduction on risk of cholelithiasis after
bariatric surgery. Obes Surg.

[4] Bastouly M, Arasaki CH, Ferreira JB, Zanoto A, Borges FGH, Del Grande JC (2009). Early changes in postprandial gallbladder emptying in morbidly
obese patients undergoing Roux-en-Y gastric bypass correlation with the
occurrence of biliary sludge and gallstones. Obes Surg.

[5] Younes S, Rizzotto MLF, Araújo ACF (2017). Itinerário terapêutico de pacientes com obesidade atendidos em
serviço de alta complexidade de um hospital universitário. Saúde Debate.

[6] Buchwald H (2005). Consensus conference statement bariatric surgery for morbid
obesity: health implications for patients, health professionals, and
third-party payers. Surg Obes Relat Dis.

[7] Patel JA, Patel NA, Piper GL, Smith DE, Malhotra G, Colella JJ (2009). Perioperative management of cholelithiasis in patients presenting
for laparoscopic Roux-en-Y gastric bypass have we reached a
consensus?. Am Surg.

[8] Warschkow R, Tarantino I, Ukegjini K, Beutner U, Güller U, Schmied BM, Müller SA, Schultes B, Thurnheer M (2013). Concomitant cholecystectomy during laparoscopic Roux-en-Y gastric
bypass in obese patients is not justified a meta-analysis. Obes Surg.

[9] Desbeaux A, Hec F, Andrieux S, Fayard A, Bresson R, Pruvot M-H, Mulliez E (2010). Risk of biliary complications in bariatric
surgery. J Visc Surg.

[10] Wudel LJ, Wright JK, Debelak JP, Allos TM, Shyr Y, Chapman WC (2002). Prevention of gallstone formation in morbidly obese patients
undergoing rapid weight loss results of a randomized controlled pilot
study. J Surg Res.

[11] Iorgulescu A, Turcu F, Iordache N (2014). ERCP after bariatric surgery--literature review and case
report. J Med Life.

[12] Mutignani M, Marchese M, Tringali A, Tacchino RM, Matera D, Foco M, Greco F, Costamagna G (2007). Laparoscopy-assisted ERCP after biliopancreatic
diversion. Obes Surg.

[13] Oana S, Shibata S, Matsuda N, Matsumoto T (2015). Efficacy and safety of double-balloon endoscopy-assisted
endoscopic papillary large-balloon dilatation for common bile duct stone
removal. Dig Liver Dis.

[14] Uy MC, Talingdan-Te MC, Espinosa WZ, Daez ML, Ong JP (2008). Ursodeoxycholic acid in the prevention of gallstone formation
after bariatric surgery a meta-analysis. Obes Surg.

[15] Worobetz LJ, Inglis FG, Shaffer EA (1993). The effect of ursodeoxycholic acid therapy on gallstone formation
in the morbidly obese during rapid weight loss. Am J Gastroenterol.

[16] Taylor J, Leitman IM, Horowitz M (2006). Is routine cholecystectomy necessary at the time of Roux-en-Y
gastric bypass. Obes Surg.

[17] Stokes CS, Gluud LL, Casper M, Lammert F (2014). Ursodeoxycholic acid and diets higher in fat prevent gallbladder
stones during weight loss a meta-analysis of randomized controlled
trials. Clin Gastroenterol Hepatol.

[18] Tolman KG, Dalpiaz AS (2007). Treatment of non-alcoholic fatty liver disease. Ther Clin Risk Manag.

[19] Cho T, Kim YJ, Paik SS (2012). The efficacy of pharmacological treatment in pediatric
nonalcoholic Fatty liver disease. Pediatr Gastroenterol, Hepatol Nutr.

[20] Le TA, Loomba R (2012). Management of non-alcoholic fatty liver disease and
steatohepatitis. J Clin Exp Hepatol.

[21] Vajro P, Lenta S, Pignata C, Salerno M, D'Aniello R, De Micco I, Paolella G, Parenti G (2012). Therapeutic options in pediatric non alcoholic fatty liver
disease current status and future directions. Ital J Pediatr.

[22] Higuera-de la Tijera F, Servin-Caamano AI (2015). Pathophysiological mechanisms involved in non-alcoholic
steatohepatitis and novel potential therapeutic targets. World J Hepatol.

