# Erratum

**DOI:** 10.1590/s0102-865020190010000009erratum

**Published:** 2019-05-06

Manuscript: Ursodeoxycholic acid in the prevention of gallstones in patients subjected to Roux-en-Y gastric bypass

Publication: Acta Cir Bras. 2019;34(1): e20190010000009

DOI: http://dx.doi.org/10.1590/s0102-865020190010000009

On Title Page of the original publication, instead of:

Francisco Heine Ferreira Machado^I^, Heladio Feitosa de Castro Filho^II^, Rodrigo Feitosa de Albuquerque Lima Babadopulos^III^, Hermano Alexandre Lima Rocha^IV^, José Lima de Carvalho Rocha^V^, Manoel Odorico de Moraes Filho^VI^

^I^PhD, Department of Surgery, Universidade Federal do Ceará (UFC), Fortaleza-CE, Brazil. Intellectual, conception and design of the study, critical revision, final approval. 

^II^MD, Department of Surgery, UFC, Fortaleza-CE, Brazil. Analysis and interpretation of data, final approval.

^III^MD, Hospital Geral Dr César Cals de Oliveira, Fortaleza-CE, Brazil. Analysis and interpretation of data, final approval.

^IV^PhD, Community Health Department, UFC, Fortaleza-CE, Brazil. Analysis and interpretation of data, final approval.

^V^PhD, UNICHRISTUS, Fortaleza-CE, Brazil. Analysis and interpretation of data.

^VI^PhD, Full Professor, Department of Farmacology, UFC, Fortaleza-CE, Brazil. Manuscript writing, critical revision, final approval.

Consider this:

Francisco Heine Ferreira Machado^I^, Heladio Feitosa de Castro Filho^II^, Rodrigo Feitosa de Albuquerque Lima Babadopulos^III^, Hermano Alexandre Lima Rocha^IV^, Maria da Conceição Cavalcante Costa^V^, José Lima de Carvalho Rocha^VI^, Manoel Odorico de Moraes Filho^VII^

^I^PhD, Department of Surgery, Universidade Federal do Ceará (UFC), Fortaleza-CE, Brazil. Intellectual, conception and design of the study, critical revision, final approval. 

^II^MD, Department of Surgery, UFC, Fortaleza-CE, Brazil. Analysis and interpretation of data, final approval.

^III^MD, Hospital Geral Dr César Cals de Oliveira, Fortaleza-CE, Brazil. Analysis and interpretation of data, final approval.

^IV^PhD, Community Health Department, UFC, Fortaleza-CE, Brazil. Analysis and interpretation of data, final approval.

^V^Master, Serviço de Atendimento Móvel de Urgência (SAMU), Fortaleza-CE, Brazil. Analysis and interpretation of data.

^VI^PhD, UNICHRISTUS, Fortaleza-CE, Brazil. Analysis and interpretation of data.

^VII^PhD, Full Professor, Department of Farmacology, UFC, Fortaleza-CE, Brazil. Manuscript writing, critical revision, final approval.

